# Combining non-microbial biostimulants and different application methods affects yield, antioxidant activity, nutritional and functional value of pea and arugula microgreens

**DOI:** 10.3389/fpls.2026.1913861

**Published:** 2026-08-31

**Authors:** Beppe Benedetto Consentino, Pietro Bellitto, Fabiana Mancuso, Francesco Salamone, Theodora Ntanasi, Georgia Ntatsi, Simona Napoli, Filippo Sgroi, Salvatore La Bella, Leo Sabatino

**Affiliations:** 1 Department of Agricultural, Food and Forest Sciences, University of Palermo, Palermo, Italy; 2 Laboratory of Vegetable Production, Department of Crop Science, Agricultural University of Athens, Athens, Greece; 3 Department of Economics, Business and Statistics, University of Palermo, Palermo, Italy; 4 Research Consortium for the Development of Innovative Agro-Environmental Systems (Corissia), Palermo, Italy

**Keywords:** *Eruca sativa*, *Pisum sativum*, priming, protein hydrolisate, root application, seaweed extract

## Abstract

Microgreens are considered a good source of nutritional and functional compounds, offering humans significant health benefits. Biostimulants are recognized as a sustainable tool to elicit growth and development of vegetables. The aim of this research was to evaluate the effects of a plant protein hydrolysate (PH) and a seaweed extract (SWE) – supplied via seed priming or root application – on pea and arugula microgreens performance. Moreover, a partial budget analysis was conducted to assess the economic implication of biostimulants application. Hydropriming and PH-priming treatments enhanced pea microgreens yield compared to the root application treatment’s counterpart by 53.2% and 33.3%, respectively. Nitrates were increased by SWE application and reduced by PH supply, in both species, compared with the not biostimulated plants, particularly when biostimulants were applied via root application [SWE, + 26.6% (pea); PH, -19.9% (pea), SWE, + 6.5% (arugula); PH, -24.3% (arugula)]. The combination of biostimulants and priming enhanced ascorbic acid compared to the control × root application (negative control) in pea microgreens by 20.9%, whereas, in arugula, either the biostimulants or priming alone significantly improved the aforesaid parameter. Biostimulants boosted total polyphenol content in pea microgreens, supplied via priming (SWE, + 21.5%; PH, 22.8%, compared to the hydropriming) or root application (SWE, + 15.7%; PH, 14.6%, compared with the negative control). Total polyphenols in arugula microgreens increased when PH and SWE were supplied via priming (+14.3%, for both biostimulants, compared to the hydropriming). Biostimulants significantly increased the antioxidant capacity (DPPH), although the method of application did not significantly affect DPPH. These findings confirmed the efficacy of biostimulants in modulating microgreens qualitative features. Furthermore, the partial budget analysis underlined that the use of both biostimulants, independently of the type of application, is economically suitable for pea, but not always convenient for arugula. The application of PH or SWE, especially via root application, was a suitable strategy to increase the nutritive and functional values of pea and arugula microgreens, contributing to their potential as valuable novel foods for human consumption.

## 1 Introduction

The growing demand for healthy diet/dishes has progressively increased the search for novel promising food products (Pallazola et al., 2019; Cena and Calder, 2020; Vasto et al., 2023). Microgreens are young and tender seedlings obtained by growing the seeds of various species of vegetables in greenhouse or in indoor cultivation systems (Tomas et al., 2021; Dereje et al., 2023). They are generally harvested at the first true leaf stage when plantlets have reached 3 to 9 cm in height. As reported by different authors (Kyriacou et al., 2016; Li et al., 2021), microgreens cultivation can be performed in different environments and growing systems, based on the scale of production. Furthermore, although the use of fertigation is not mandatory, it is highly recommended to enhance microgreens productive and qualitative features (Li et al., 2023). Even though microgreens are not considered substitutes for their relative standard vegetables, they represent a novel sector of the edible vegetable industry. They can offer a great variety of flavors, textures and colors to improve and decor main and second side dishes, sandwiches, salads and appetizers (Bhaswant et al., 2023). Indeed, microgreens are also defined as superfoods due to their higher content of phytochemicals compared to the mature vegetable counterpart (Rizvi et al., 2023). For instance, there is evidence of higher nutritive and functional components in pea (*Pisum sativum* L.) and arugula (*Eruca sativa* Mill.) microgreens compared to their mature counterparts (Drygaś et al., 2024; Pérez-Leal et al., 2025). According to Balik et al. (2025) and Komeroski et al. (2023), pea and arugula microgreens exhibit high content of carotenoids, chlorophylls, and bioactive compounds, underlining their important antioxidant activity in human body system.

The European regulation (European Green Deal strategies) recognizes biostimulants as sustainable tools to elicit growth and development of vegetables (Consentino et al., 2021; Consentino et al., 2022; Di Miceli et al., 2023; Vultaggio et al., 2024). Biostimulants, regardless of their origin, enhance plant resilience to various adverse agro-environments by stimulating primary metabolism, behaving like physiological primers due to their hormone-like activity, and modifying antioxidant systems (Colla et al., 2017; Masondo et al., 2018; Ciriello et al., 2022). They can be categorized based on their nature (microbial or non-microbial), formulation (powder, liquid, and granular), and application techniques (Gupta et al., 2021). Although biostimulants are mostly supplied via foliar spray or fertigation (Colla et al., 2015a), they could be also applied to seeds via coating or priming methods. These methods might be also applied to microgreens production (de la Fuente et al., 2019; Rai et al., 2022) which, as above mentioned, represent a new specialty crop in the agri-food sector. Although there are scientific studies focusing on different aspects of microgreens cultivation such as the type of plant material (de la Fuente et al., 2019), substrate choice (Du et al., 2022), nutrient solution management (Kyriacou et al., 2021) and control of climatic parameters (Zhang et al., 2020), a few studies have been conducted on the effects of biostimulants on microgreens seed and seedlings growth. In this respect, there are evidences that non-microbial biostimulants (animal-, plant-, or microalga-based biostimulant) elicit germination and hypocotyl elongation in different species (Pannacci et al., 2022; Karbarz et al., 2023). Although, Ciriello et al. (2023), found that a priming treatment with biostimulants boosts nutraceutical characteristics in Mibuna and Komatsuna microgreens, nevertheless, the influences of microbial and/or non-microbial biostimulants applied on seeds and seedlings growth are still understudied. Current studies focus on investigating the application of biostimulants via a single approach (radical or priming), without ever comparing the different application strategies (Ciriello et al., 2023; Drygaś et al., 2024; Pérez-Leal et al., 2025). This study aims to fill this gap to provide information on the effects of biostimulants and their application methods on pea and arugula microgreens.We assume that the microgreens short crop cycle could destabilize biostimulant effects. Likewise, biostimulants supply via foliar spray might result in mechanical injury to young seedlings and final product contamination. Since the signaling molecules enclosed in biostimulants might have a different effect on plant performance depending on the application mode, our hypothesis was that the biostimulant type of application could differently modulate microgreens performance and that there might be an optimal application mode for each class of biostimulants depending on the target (yield or quality). In this regard, seed priming acts during the early stages of plant development, stimulating the activation of the metabolic processes involved in seed germination and secondary metabolism activity, thereby providing a significant advantage in terms of growth and the subsequent uptake of water and nutrients (Masondo et al., 2018). Conversely, the application of biostimulants after emergence might be more effective in promoting plant nutrient uptake and primary metabolism. Although both application methods share the common benefit of improved plant development, they may differ in terms of the accumulation of antioxidant compounds and mineral elements. Consequently, the aim of our research was to test the effects of two biostimulant categories (a commercial protein hydrolysate Tyson^®^ and a commercial seaweed extract Kelpstar^®^), applied either as priming agents or via root application, on yield, antioxidant activity, nutritional and functional value, as well as the added net return of pea and arugula microgreens. These scientific approaches could represent an imperative landmark for an eco-friendly microgreen production protocol.

## 2 Materials and methods

### 2.1 Experimental field and growth conditions

The experiment was carried out in 2024, at the Department of Agricultural, Food and Forest Sciences (SAAF—University of Palermo, Italy) (38°6’28” N, 13°21’3” E; altitude 49 m). A controlled-environment growth room was equipped with a metal shelving unit, in which LED lamps (Cosmorrow^®^ LED 40W 24V BLOOMING, by Secret Jardin) were installed on top in series (870 x 32 x 145 mm). In order to supply the visible light spectrum range, a LED lighting system was arranged (40W power LEDs), endowed with a blue led light of (440 nm) and red led light of (660 nm). The blue light peak makes it compatible with cuttings and growth. As suggested by other authors (Kyriacou et al., 2019; Li et al., 2023), LEDs provided a photosynthetic photon flux density (PPFD) of 300 ± 15 µmol m-2 s^-1^. Photoperiod conditions were settled at16 h light and 8 h dark, whereas the air temperature and relative humidity were set at 18 ± 1 °C and 70 ± 10%, respectively.

### 2.2 Plant material and treatments

Pea (*Pisum sativum* L.), cultivar Viridios (Italian Sprout, Cesena, Italy) and arugula (*Eruca sativa* Mill.), cultivar Matisse (Italian Sprout, Cesena, Italy) were tested. The effect of two biostimulants [a seaweed extract (SWE) and a protein hydrolysate (PH)] administered via two different application methods, priming or root application, were evaluated. Control plots were also included (not primed and water supply via root application). The PH (Tyson^®^, Mugavero fertilizers, Palermo, Italy) is rich in nitrogen-based substances derived totally from amino acids (see **Supplementary Table 1** for aminogram) and plant peptides (31%), obtained through enzymatic hydrolysis of soybean plants at low temperature. It is composed of 5% total nitrogen (4.5% organic nitrogen) and 25% organic carbon. The SWE (Kelpstar^®^, Mugavero fertilizers, Palermo, Italy) was produced via a cold micronization process and contained organic nitrogen and carbon (1% and 10%, respectively), phytohormones (11 mg L^-1^ of auxin and 0.03 mg L^-1^ of cytokinin), amino acids, such as glycine (0.01 g/a00 g), aspartic acid (0.01 g/100 g), glutamic acid (0.02 g/100 g), alanine (0.01 g/100 g), proline(0.01 g/100 g), and organic matter (22.5%). Priming and root application treatments with biostimulants were applied at a dose of 3 ml L^-1^ according to manufacturer recommendations. Regarding priming treatments, five grams of arugula and 35 g of pea seeds, respectively, were soaked in three different priming solutions of 40 ml [distilled water (hydropriming), PH and SWE] for 24 hours in dark at 20 °C in a rotary shaker operating at 50 rpm, providing continuous aeration of the priming solution. The seed-to-solution ratio was 1:8 (w/w) for arugula and 1:1.14 (w/w) for pea. To guarantee the absence of external contaminations, petri dishes were secured with parafilm (Parafilm^®^ M, Sigma-Aldrich, Milan, Italy). After priming and before sowing, arugula and pea seeds were washed with distilled water and restored to their original weight using a desiccator containing silica gel. At sowing, seeds were manually placed in plastic trays (16 x 11 x 4 cm) containing a peat-based mixture characterized by pH 3.5 and N, P_2_O_5_ and K_2_O content < 50 mg L^-1^ (Special Mixture, Floragard Vertriebs-GmbH, Oldenburg, Germany). From sowing until harvest, seedling were weekly watered. In addition, root application treatments, via bottom watering method, started one week after the sowing and were conducted every two days until harvest (5 applications in total), supplying 100 ml of solution for each plastic tray. Moreover, trays fertigated with biostimulants received a solution with 0.3% of PH or SWE. The theses treated with biostimulants via root application did not receive priming treatment. Non biostimulated microgreens received only water for the entire cultivation cycle. Microgreens were harvested 15 days after sowing.

### 2.3 Experimental design and statistics

For each species, two types of application (T) (priming or root application) and two different biostimulant treatments (B) (PH and SWE), along with the control (negative control) and hydropriming (positive control), were arranged in a randomized complete block design with two experimental factors (biostimulant and application method). Due to the cross combination of different biostimulant and application methods, there were six treatments. Each treatment was replicated 3 times, totalizing 18 experimental plots. Prior to statistical analysis, data were tested for normality and homoscedasticity, via Shapiro-Wilk and Levene tests (**Supplementary Tables 2**, **3**), respectively.

A combined analysis of variance (ANOVA) was used to test the effects T, B and their interaction (TxB) on yield, dry matter, mineral profile, functional traits, total sugars, pigments and antioxidant activity. When significant, differences among mean values of treatments were compared (p-value ≤ 0.05) using HSD. The analysis was performed by using SPSS software 20.0 version (StatSoft, Inc., Chicago, IL, USA).

### 2.4 Yield, dry matter content and mineral profile

At the cotyledonary stage, pea and arugula microgreens were harvested using sterilized scissors at the substrate level. After harvesting, yield was recorded and expressed as mg cm^-2^. Dry matter percentage (%) was evaluated using 50 g of fresh biomass for each replicate, samples were placed in a ventilated oven at 70 °C for 24 h and then at 105 °C for 24 h until a constant weight. The P content was evaluated via Fogg and Wilkinson (1958) colorimetric method. The K, Ca and Mg concentration on pea and arugula microgreens was assessed via atomic absorption spectroscopy following wet mineralization and employing wavelengths of 766.5 nm for K, 422.7 nm for Ca, and 285.2 nm for Mg (Nerdy, 2018). Mineral contents of microgreens (P, K, Ca and Mg) were reported as g kg-1 dw. To evaluate nitrate content, Formisano et al. (2021) method was followed. Briefly, 0.25 g dried and ground microgreens sample was used for extraction in ultrapure water and analyzed using ion chromatography. Values were reported as mg kg-1 fw.

### 2.5 Functional traits, total sugars, pigments and antioxidant activity (DPPH)

The Reflectometer Merck RQflex10 Reflectoquant^®^ and Reflectoquant Ascorbic Acid Test Strips were used to evaluate the ascorbic acid content (mg g-1 fw) in pea and arugula. To appreciate the total phenols content, reported as mg GAE g^-1^ dw, the Folin-Ciocâlteu assay was carried out (Meda et al., 2005). Vegetal samples were mixed with water, sodium carbonate (Na_2_CO3) and the Folin-Ciocâlteu reagent. After 30 minutes at 24 °C the absorbance was established at 750 nm. To quantify flavonoids content in microgreens, Meda et al. (2005) methodology was adopted, using quercetin (Sigma–Aldrich Chemie, Steinheim, Germany) as standard. The absorbance was measured at 415 nm. Flavonoids were expressed as mg quercetin g^-1^ dw. To evaluate total sugar (mg g^-1^ dw) content in pea and arugula, the method described by Serna et al. (2013) was followed. Pigments amount (chlorophylls and carotenoids) in samples were evaluated following Costache et al. (2012). The values were reported as mg 100 g^-1^ fw. Moreover, chlorophylls and carotenoids ratio were calculated. To estimate the antioxidant capacity of microgreens, DPPH scavenging (%DPPH) was established following the Chen and Ho (1997) method.

### 2.6 Partial budget analysis

A partial budget analysis was elaborated to appraise the net economic benefits that may upgrade pea and arugula microgreens production through the supply of PH and SWE via priming or root application. The economic approach described by Giordano et al. (2020) was used. The biostimulants added costs and gross returns were considered. The following formula was operated to calculate the added net return generated by PH and SWE applied via priming or root application:


Added net return=added gross return−addedd variable costs

Labor costs were obtained through direct surveys of local farmers to best reflect the operational context. The price of the biostimulants was provided by the manufacturer (Mugavero Fertilizers). The analysis presented in this study relates to the experimental context and local conditions; therefore, the transferability of the results to other contexts depends on variations in input costs, farm conditions and local market prices. Lastly, the added net return was graphically represented.

## 3 Results

### 3.1 Yield, dry matter and minerals concentration

**Figure 1:**
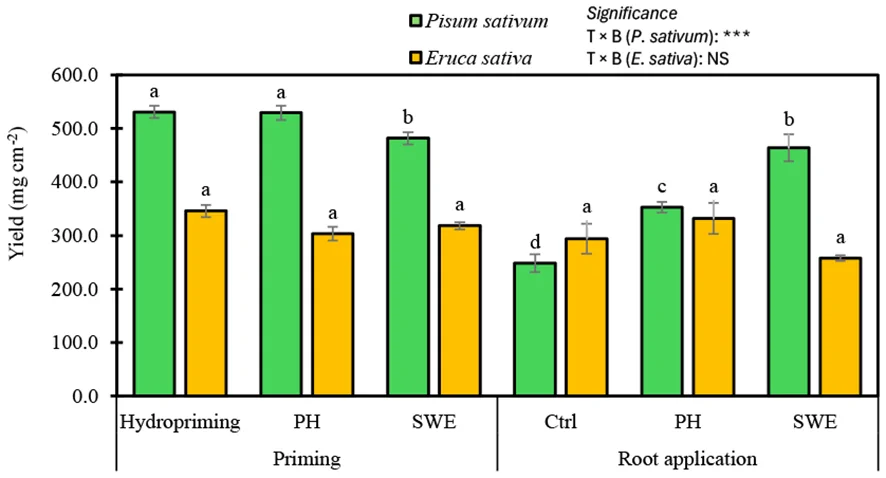
Impact of biostimulant and type of application on *Pisum sativum* and *Eruca sativa* yield. Statistical analysis was separately performed for each species. Means with different letters are significantly dissimilar according to Tukey test (*P* ≤ 0.05). NS: not statistically significant; ***: significant at P ≤ 0.001. Data is presented as mean ± SE. T, type of application; B, biostimulant; Ctrl, control; Hydropriming: hydro-primed seeds; PH, protein hydrolysate; SWE, seaweed extract.

Pea microgreens’ yield was significantly affected by the combination of biostimulants and type of application, contrariwise, no statistically significant effect was recorded in arugula yield (**Figure 1**).

Hydropriming and PH treatments gave the highest yield (530.9 and 529.3 mg cm^-2^, respectively), followed by SWE priming (**Figure 1**). The lowest yield was observed in control microgreens.

As shown in **Table 1**, *P. sativum* dry matter content was only affected by the type of application, with the highest values recorded in the root application treatments (+7.7% than priming).

**Table 1:** Impact of biostimulant and type of application on *Pisum sativum* and *Eruca sativa* dry matter content, P, K, Ca and Mg concentrations. .

Treatments		Dry matter content (%)				P (g kg^-1^ dw)				K (g kg^-1^ dw)				Ca (g kg^-1^ dw)				Mg (g kg^-1^ dw)			
		*Pisum sativum*		*Eruca sativa*		*Pisum sativum*		*Eruca sativa*		*Pisum sativum*		*Eruca sativa*		*Pisum sativum*		*Eruca sativa*		*Pisum sativum*		*Eruca sativa*	
Type of application (T)																					
Priming		8.4	b	7.2	a	7.91	b	6.56	b	24.49	b	24.79	b	5.32	a	9.32	b	3.30	a	3.34	b
Root application		9.1	a	6.8	a	8.37	a	6.95	a	25.12	a	25.73	a	5.23	a	9.63	a	3.34	a	3.49	a
Biostimulant (B)																					
Ctrl		8.6	a	7.8	a	7.83	b	6.59	b	24.07	b	24.82	b	5.14	a	9.27	b	3.31	a	3.36	b
PH		9.0	a	6.4	b	8.28	a	6.87	a	25.10	a	25.43	a	5.39	a	9.57	a	3.32	a	3.42	ab
SWE		8.8	a	6.8	ab	8.30	a	6.81	ab	25.25	a	25.53	a	5.30	a	9.59	a	3.32	a	3.47	a
*Type of application*	*Biostimulant*																				
Priming	Hydropriming	8.6	a	8.8	a	7.83	b	6.52	a	24.03	a	24.8	b	5.29	a	9.3	b	3.37	a	3.4	b
	PH	8.1	a	6.8	bc	7.90	b	6.61	a	24.77	a	24.7	b	5.42	a	9.2	b	3.26	A	3.3	b
	SWE	8.6	a	6.2	c	8.00	b	6.56	a	24.67	a	24.9	b	5.25	a	9.4	b	3.25	A	3.4	b
Root application	Ctrl	8.6	a	6.8	bc	7.83	b	6.66	a	24.10	a	24.8	b	4.99	a	9.2	b	3.25	a	3.4	b
	PH	9.8	a	6.1	c	8.67	a	7.13	a	25.43	a	26.2	a	5.36	a	9.9	a	3.37	a	3.5	a
	SWE	9.0	a	7.5	b	8.60	a	7.05	a	25.83	a	26.2	a	5.35	a	9.8	a	3.39	a	3.6	a
Significance																					
T		*		NS		***		***		**		***		NS		***		NS		***	
B		NS		*		***		*		***		**		NS		**		NS		*	
T × B		NS		**		***		NS		NS		**		NS		**		NS		**	

Statistical analysis was separately performed for each species. Means with different letters are significantly dissimilar according to Tukey test (*P* ≤ 0.05). NS: not statistically significant; *: significant at P ≤ 0.05; **: significant at P ≤ 0.01; ***: significant at P ≤ 0.001. Data is presented as mean ± SE. T, type of application; B, biostimulant; Ctrl, control; Hydropriming, hydro-primed seeds; PH, protein hydrolysate; SWE, seaweed extract.

*E. sativa* dry matter content was modulated by the treatments’ interaction with the highest values recorded in microgreens subjected to hydropriming (8.8%). The lowest dry matter content was found in microgreens primed with SWE and in those treated with PH via root application (**Table 1**). As reported in **Table 1**, the P concentration in pea microgreens was affected by the treatment’s interaction. Specifically, the highest values were found in plants supplied with PH or SWE via root application (8.67 and 8.60 g kg^-1^ dw, respectively), whereas all the other treatment combinations gave the lowest P content. For arugula P concentration, statistical analysis revealed no significant interaction between treatments. The root application treatment, independently of the biostimulant application, significantly enhanced P concentration (+5.5% than priming), whereas, non-considering the type of application, the use of PH gave the highest P values (6.87 g kg^-1^ dw), along with SWE. The K concentration in pea microgreens was not affected by main factors’ interaction (**Table 1**). Both the root application treatment and the biostimulants application (PH or SWE) significantly enhanced K values. For *E. sativa* K concentration, a significant interaction among treatments (T × B) was recorded, following the same trend as described for *P. sativum* P concentration. Pea Ca and Mg concentrations were not affected by the treatments, whereas ANOVA for arugula data showed a significant treatment interaction (**Table 1**). The highest Ca and Mg concentrations were assessed in microgreens treated with PH (+7.1% and +2.8% for Ca and Mg, respectively) or SWE (+6.1% and +5.5% for Ca and Mg, respectively) via root application.

### 3.2 Nitrate, ascorbic acid, total polyphenols, flavonoids and total sugars

**Figure 2:**
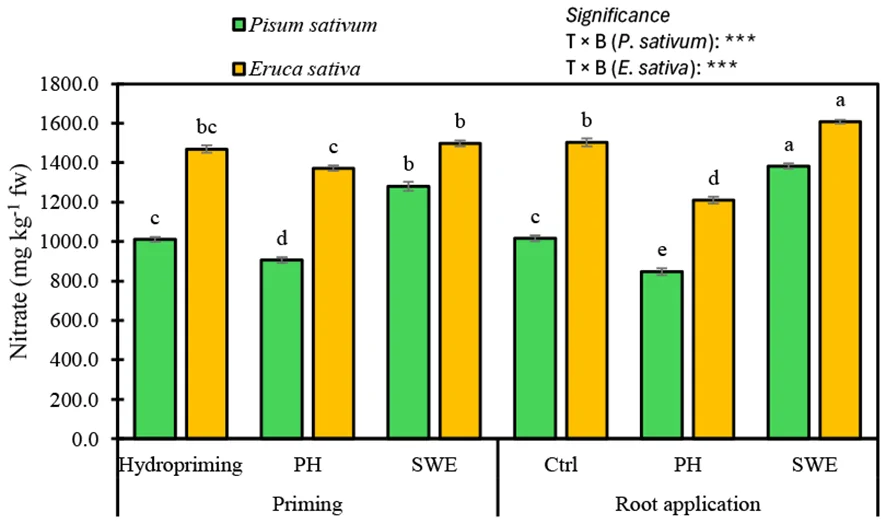
Impact of biostimulant and type of application on *Pisum sativum* and *Eruca sativa* nitrate concentration. Statistical analysis was separately performed for each species. Means with different letters are significantly dissimilar according to Tukey test (*P* ≤ 0.05). ***: significant at P ≤ 0.001. Data is presented as mean ± SE. T, type of application; B, biostimulant; Ctrl, control; Hydropriming, hydro-primed seeds; PH, protein hydrolysate; SWE, seaweed extract.

The type of application (T) and the biostimulant treatments (B) significantly modulated nitrate concentration in pea and arugula microgreens (**Figure 2**).

PH reduced nitrate concentration of both species by -10.9% and -8.8% in pea and arugula, respectively as compared to the negative control, whereas SWE significantly enhanced it. The highest nitrate concentration was found when both species were supplied with SWE via root application, whereas the lowest ones were found in PH-treated plants (**Figure 2**).

**Figure 3:**
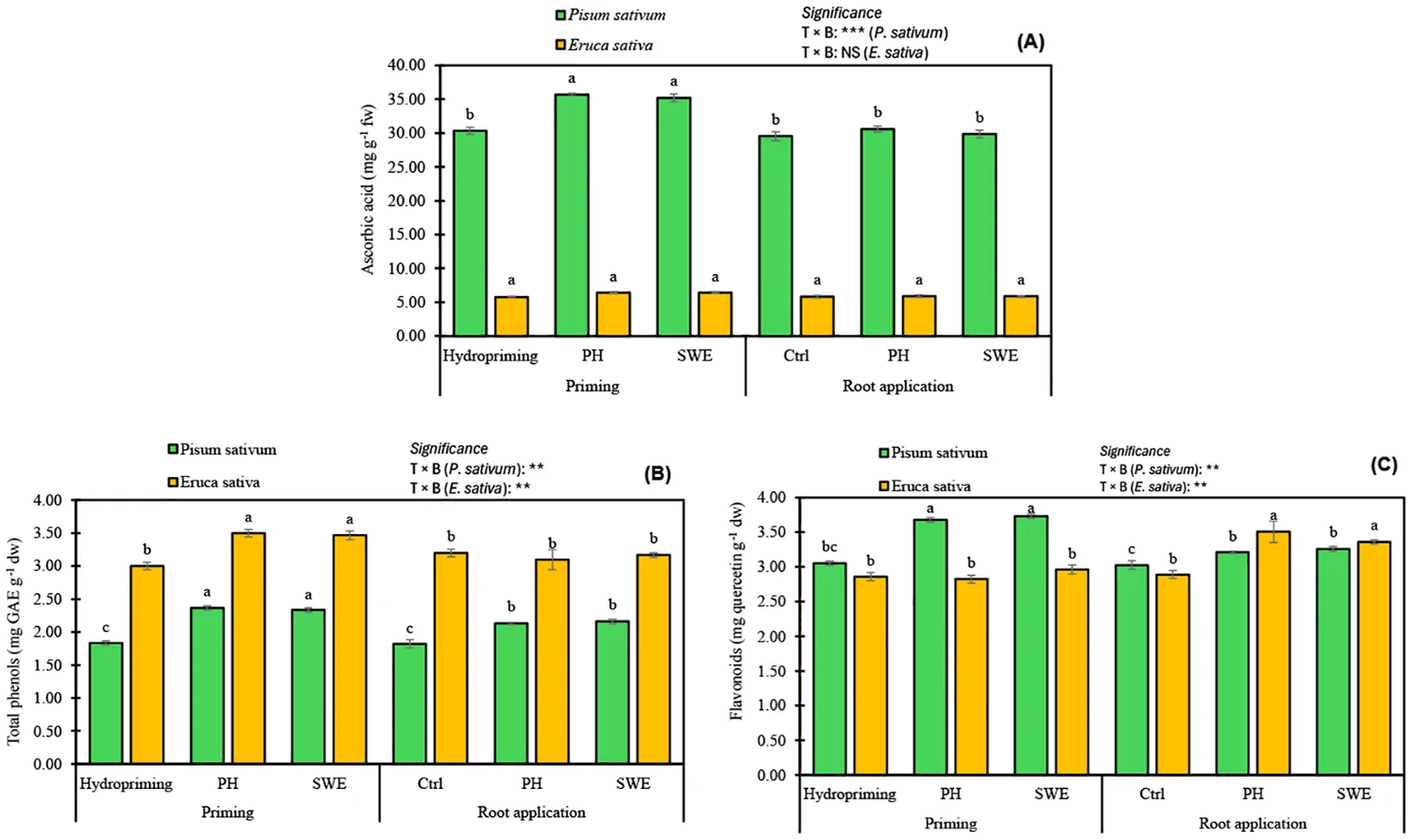
Impact of biostimulant and type of application on *Pisum sativum* and *Eruca sativa* ascorbic acid **(A)**, total phenols **(B)** and flavonoids **(C)** concentrations. Statistical analysis was separately performed for each species. Means with different letters are significantly dissimilar according to Tukey test (*P* ≤ 0.05). ***: significant at P ≤ 0.001. Data is presented as mean ± SE. T: type of application; B: biostimulant; Ctrl: control; Hydropriming: hydro-primed seeds; PH: protein hydrolysate; SWE: seaweed extract.

The main factors interaction significantly affected ascorbic acid content in pea microgreens, whereas this effect was not recorded in arugula microgreens (**Figure 3A**).

Seed priming either with PH or SWE gave the highest ascorbic acid content in *Pisum sativum* microgreens (35.70 and 35.20 mg g^-1^ fw). ANOVA, for total phenols, had a significant effect of T×B interaction (**Figure 3B**). Priming with PH or SWE significantly improved total phenols concentration by 30.2% and 28.0%, respectively as compared to the negative control in pea and by 9.4%, for both biostimulants, in arugula microgreens (**Figure 3B**). Combining the main factors significantly affected flavonoids concentration in both species (**Figure 3C**). The highest values were assessed in PH (3.68 mg quercetin g^-1^ dw) and in SWE-primed (3.73 mg quercetin g^-1^ dw) pea plants, followed by those detected in plants exposed to PH or SWE via root application. Control microgreens revealed the lowest values (3.05 and 3.03 mg quercetin g^-1^ dw for hydropriming and negative control, respectively). For arugula, the highest values were recorded in microgreens provided with both biostimulants (PH or SWE) through root application (3.51 and 3.36 mg quercetin g^-1^ dw, respectively), followed by those detected in the other treatment combinations.

**Figure 4:**
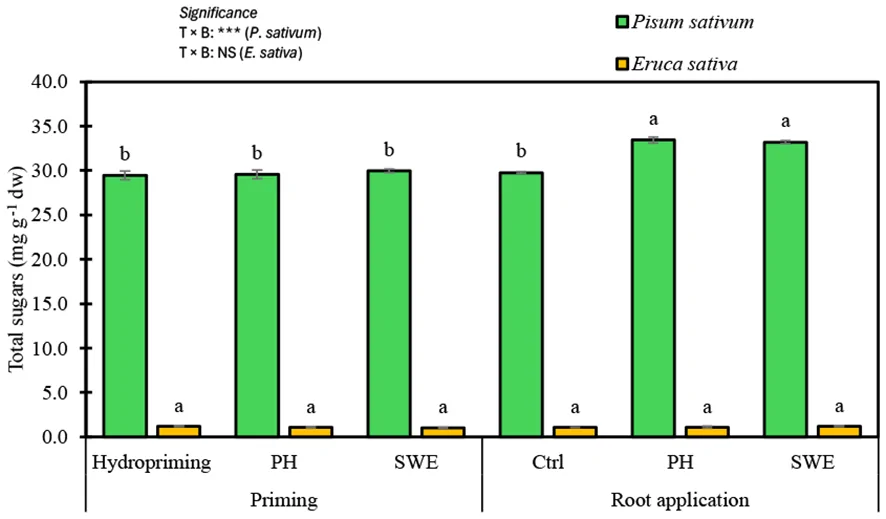
Impact of biostimulant and type of application on *Pisum sativum* and *Eruca sativa* total sugars concentrations. Statistical analysis was separately performed for each species. Means with different letters are significantly dissimilar according to Tukey test (*P* ≤ 0.05). NS: not statistically significant; ***: significant at P ≤ 0.001. Data is presented as mean ± SE. T, type of application; B, biostimulant; Ctrl, control; Hydropriming, hydro-primed seeds; PH, protein hydrolysate; SWE, seaweed extract.

Combining biostimulants and type of application significantly affects total sugars concentration in pea, but not in arugula (**Figure 4**).

The highest total sugars were found in pea microgreens supplied with PH or SWE, whereas the other treatment combinations revealed the lowest values.

### 3.3 Total chlorophyll, carotenoids, total chlorophyll/carotenoids ratio and DPPH

The T × B interaction had no effect on pea total chlorophyll, while significantly affected arugula (**Table 2**).

**Table 2:** Impact of biostimulant and type of application on *Pisum sativum* and *Eruca sativa* total chlorophyll, carotenoids, total chlorophyll/carotenoids ratio and DPPH.

Treatments		Total chlorophyll (mg 100 g^-1^ fw)				Carotenoids (mg 100 g^-1^ fw)				Total chlorophyll/carotenoits ratio				DPPH (mg tocoferol equivalent g^-1^ dw)			
		*Pisum sativum*		*Eruca sativa*		*Pisum sativum*		*Eruca sativa*		*Pisum sativum*		*Eruca sativa*		*Pisum sativum*		*Eruca sativa*	
Type of application																	
Priming		399.1	b	85.7	a	84.7	a	18.6	a	4.7	b	4.6	b	4.3	a	4.7	a
Root application		424.3	a	87.2	a	81.5	b	16.3	b	5.2	a	5.4	a	4.4	a	4.8	a
Biostimulant																	
Ctrl		395.8	b	80.6	b	78.8	b	17.1	a	5.0	a	4.7	b	4.2	b	4.6	b
PH		420.1	a	89.4	a	86.0	a	17.8	a	4.9	a	5.1	ab	4.5	a	4.9	a
SWE		419.3	a	89.4	a	84.5	a	17.5	a	5.0	a	5.2	a	4.4	a	4.9	a
*Type of application*	*Biostimulant*																
Priming	Hydropriming	383.2	a	82.3	b	79.6	a	17.5	b	4.8	a	4.7	b	4.2	a	4.7	a
	PH	407.7	a	87.3	ab	87.7	a	19.4	a	4.7	a	4.5	b	4.4	a	4.8	a
	SWE	406.4	a	87.6	ab	86.8	a	19.1	a	4.7	a	4.6	b	4.4	a	4.8	a
Root application	Ctrl	408.3	a	78.9	c	78.0	a	16.7	bc	5.2	a	4.7	b	4.1	a	4.6	a
	PH	432.5	a	91.6	a	84.2	a	16.2	c	5.1	a	5.7	a	4.6	a	5.0	a
	SWE	432.3	a	91.2	a	82.3	a	16.0	c	5.3	a	5.7	a	4.5	a	5.0	a
Significance																	
T		***		NS		***		***		***		***		NS		NS	
B		***		***		***		NS		NS		*		***		**	
T × B		NS		*		NS		*		NS		**		NS		NS	

Statistical analysis was separately performed for each species. Means with different letters are significantly dissimilar according to Tukey test (*P* ≤ 0.05). NS: not statistically significant; *: significant at P ≤ 0.05; **: significant at P ≤ 0.01; ***: significant at P ≤ 0.001. Data is presented as mean ± SE. T, type of application; B, biostimulant; Ctrl, control; Hydropriming, hydro-primed seeds; PH, protein hydrolysate; SWE, seaweed extract.

As chlorophyll content, data underlined a positive effect of both main factors (**Table 2**). For *E. sativa*, the highest concentrations were assessed in microgreens treated with PH or SWE via root application (91.6 and 91.2 mg 100 g^-1^ fw, respectively), followed by those hydroprimed. The lowest total chlorophyll was noted in control microgreens. The interaction between the two main factors did not influence *P. sativum* carotenoids content, whereas affected *E. sativa* (**Table 2**). Either priming and biostimulants significantly enhanced carotenoids concentration in pea microgreens, compared to other treatments. Carotenoids peaked in arugula microgreens primed with PH or SWE (19.4 and 19.1 mg 100 g^-1^ fw, respectively), whereas microgreens provided with PH or SWE through root application gave the lowest values. Total chlorophyll/carotenoids ratio was affected only by the type of application in *P. sativum*, while it was influenced by the interaction between treatments in *E. sativa* (**Table 2**). Root application meaningfully enhanced total chlorophyll/carotenoids ratio in pea microgreens, whereas for arugula the peak was recorded in microgreens primed with PH or SWE. The DPPH values were not affected by the treatment’s interaction in both species. The type of application did not significantly influence the DPPH values in both species, whereas, not considering the type of application, the use of both biostimulants increased the DPPH compared to the control treatment.

### 3.4 Partial budget analysis

**Figure 5:**
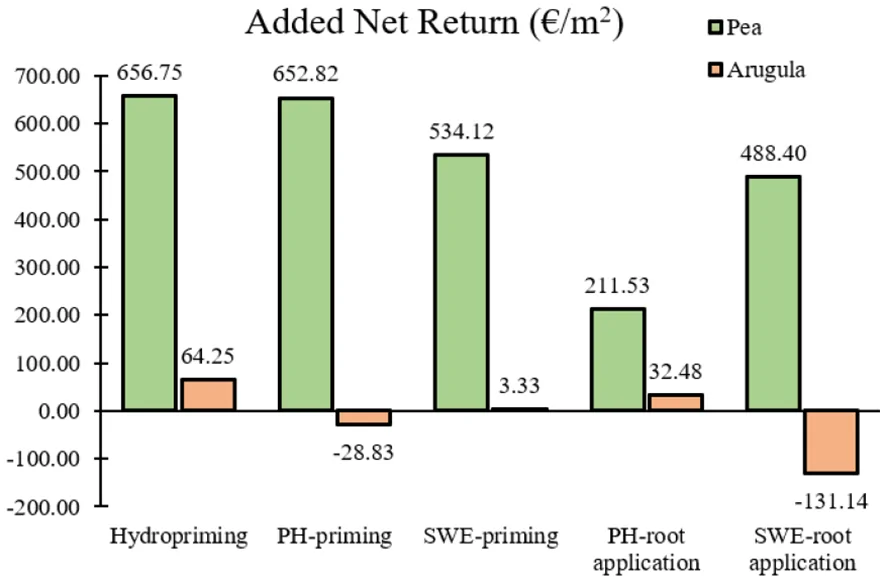
Graphical representation of pea and arugula added net return in response to biostimulants application via priming or root application.

For pea microgreens production, the economic appraisal underlined a beneficial impact of the hydropriming (656.75 EUR/m^2^) and the PH priming (652.82 EUR/m^2^). However, the other treatments significantly increased the added net return, 534.12 EUR/m^2^, 211.53 EUR/m^2^ and 488.40 EUR/m^2^, for SWE priming, PH root application and SWE root application, respectively (**Table 3**, **Figure 5**).

**Table 3:** Added net return for pea microgreens achieved by the application protein hydrolysate (PH) or seaweed extract (SWE) via priming or root application.

Biostimulant	Yield increase (kg m^-2^)	Price (€ kg^-1^)	Added gross return (€ m^-2^)	Added variable cost (€ m^-2^)				Added net return (€ m^-2^)
				Biostimulant treatment	Application	Harvest	Total	
Hydropriming	2,8	250	706,8	0	50	0	50	656,75
PH_priming	2,8	250	702,9	0,10	50	0	50,1	652,82
SWE_priming	2,3	250	584,3	0,13	50	0	50,13	534,12
PH_root application	1,1	250	262,6	1,05	50	0	51,05	211,53
SWE_root application	2,2	250	539,8	1,35	50	0	51,35	488,40

SWE: 13.5 EUR/L; PH: 10.5 EUR/L.

Regarding the arugula microgreens, the hydropriming treatment gave the highest add net return (64.25 EUR/m^2^), followed by PH root application (32.48 EUR/m^2^). Whereas negative values were observed when microgreens were exposed to PH priming (-28.83 EUR/m^2^) or SWE root application (-131.14 EUR/m^2^) (**Table 4**, **Figure 5**).

**Table 4:** Added net return for arugula microgreens achieved by the application protein hydrolysate (PH) or seaweed extract (SWE) via priming or root application.

Biostimulant	Yield increase (kg m^-2^)	Price (€ kg^-1^)	Added gross return (€ m^-2^)	Added variable cost (€ m^-2^)				Added net return (€ m^-2^)
				Biostimulant treatment	Application	Harvest	Total	
Hydropriming	0,5	220	114,25	0	50	0	50	64,25
PH_priming	0,1	220	21,27	0,10	50	0	50,1	-28,83
SWE_priming	0,2	220	53,46	0,13	50	0	50,13	3,33
PH_root application	0,4	220	83,53	1,05	50	0	51,05	32,48
SWE_root application	-0,4	220	-79,79	1,35	50	0	51,35	-131,14

SWE: 13.5 EUR/L; PH: 10.5 EUR/L.

## 4 Discussion

The growing interest in novel superfood, such as microgreens, useful to implement the nutritional status of humans, has increased the search for new cultivation schemes to enhance yield and nutritional properties. The purpose of this study was to appraise the effects of two biostimulants (a plant protein hydrolysate and a seaweed extract) administered via priming or root application on two plant species grown as microgreens (*Pisum sativum* and *Eruca sativa*). Pea yield was increased by seed priming and by biostimulants compared to the control. These data agree with those of Çakır et al. (2025). who reported an increase in yield of parsley microgreens subjected to priming, compared to non-primed plants. The increase of yield in pea can be associated with the priming treatments, which guaranteed quicker and uniform seed germination, boosting plant growth rate (Tamindžić et al., 2023). It is also reported that primed seed absorbs more water and develop stronger roots than those not primed, being ready to start a quick and healthier growth (Shah et al., 2012). The non-significant effect of priming observed on arugula microgreens yield can be ascribed to a different species seed sensitivity to seed priming (mainly for different sizes). Regarding biostimulants effect and type of application on pea, SWE primed seeds had lower yield compared to those hydro-primed or primed with PH. Both biostimulants applied by root application significantly enhanced pea yield compared to the control. Protein hydrolysates are mainly made of amino acids and peptides with hormone-like activity (Colla et al., 2017), whereas SWE are principally composed of polysaccharides, vitamins and minerals (Ali et al., 2021). Due to their composition, protein hydrolysates have been observed to promote primary metabolic processes by influencing various metabolic pathways. In particular, amino acids serve as the main transport medium for organic nitrogen in plants and provide accessible materials for protein production, which in turn supports improved plant growth and metabolic activity (Colla et al., 2017). Consequently, we assume that the compounds comprised in PH can be easily absorbed by seeds, unlike those enclosed in SWE that are more complex and difficult to be effective for seeds hydration. Data on root application gave a clearer overview of the positive effects of SWE when applied via root application encouraging our assumptions. Arugula’s lack of response can be related to its slower plant growth rate which in turn does not allow efficient action of the biostimulants. In general, differences between priming and root application can derive from the timing of the treatment itself. In fact, seeds priming activates embryonal metabolic pathways, enhancing plantlets uniformity and production of antioxidants (Ciriello et al., 2023), whereas fertilization post-emergence has a less pronounced effect on qualitative traits and overall growth. Moreover, the different response of the two microgreens species to the nutrient solution can be linked to their capacity to absorb nutrients, as well as their ability to use bioactive components included in the biostimulant solutions, enclosing those characterized by hormone-like activity.

Priming reduced dry matter content of pea microgreens, whereas it did not affect dry matter content of arugula. These data are partially in line with those of Armin et al. (2010) who found no significant effect of priming on watermelon seedlings. The reduction of dry matter content could be the consequence of the priming effect on seed germination and growth, which leads to a quicker build-up of water rather than carbohydrates or other substances that constitute the plant dry matter content. At this regard, since microgreens are harvested in early development phase, even small variation in water uptake could determine variations in dry matter percentage. In the present study, biostimulants reduced arugula dry matter content and had no significant effect on pea dry matter percentage. This effect can be ascribed to the increased plant water uptake prompted by biostimulants supply (Rouphael and Colla, 2020). Moreover, the different data between arugula and pea suggested a species-related response to treatments, which in turn can be explained as physiological differences in water uptake and distribution during the early growth stage. Root application treatments and biostimulants application increased all pea nutrients except for Ca and Mg. Data are partially consistent with those of Ciriello et al. (2024) on brassicaceous microgreens, who found different responses based on species. Priming treatments stimulated the initial germination and plant growth but did not guarantee continuous supply later on. The stable application of biostimulants ensures that their positive effects on plant nutrition are efficiently exerted. At this regard, it was reported that biostimulants can synergize with nutrients, enhancing their solubility (Rouphael and Colla, 2020). Biostimulants like PH and SWE can ameliorate root cells permeability to nutrients, increase overall plant nutrients uptake and enhance roots elongation (Sible et al., 2021).

Nitrate concentration of both species was reduced by PH and increased by SWE applications, particularly in root application treatments, compared to the control. These results are in accordance with the findings of La Bella et al. (2021) who underline a nitrogen increase in SWE-treated spinach plants, and with those of Sabatino et al. (2021), who reported a reduction when lettuce plants were treated with PH. The different effects recorded for PH and SWE on nitrate can be ascribed to their composition. Indeed, SWE contains little amounts of nutrients and phytohormones, such as auxin and cytokinin, that stimulate plant growth and the nutrient uptake, comprising nitrogen (Ali et al., 2021); whereas PH comprises amino acids which, once absorbed, regulates nitrogen assimilation (Colla et al., 2015b). Moreover, these data suggest that biostimulants differentially modulate plant nitrogen metabolism, indeed, SWE that seems to elicit nitrogen uptake and accumulation, whereas PH had the opposite effect. The more pronounced effect of root application compared with priming indicated that the continuous availability of biostimulant on the rhizosphere enhances the effectiveness of both biostimulants.Priming significantly increased antioxidants and secondary metabolites, such as ascorbic acid, polyphenols and flavonoids. These data agree with those of Ciriello et al. (2023) who reported significant increase in secondary metabolites of primed microgreens. Plants from seeds subjected to priming tend to have higher contents of such metabolites, compared to non-primed seeds, because of several physiological and biochemical changes generated by the treatment. There is evidence that priming can stimulate the production of secondary metabolites and antioxidants via the activation of metabolic ways for combating the oxidative stress caused by the environmental conditions (Salami et al., 2023). Priming also influences hormone activity, like auxin, cytokinin and gibberellin, which in turn can stimulate the production of secondary metabolites (Zhu et al., 2021). Our data also revealed that both biostimulants significantly enhanced ascorbic acid, total phenols and flavonoids. These results were partially in line with those of El-Nakhel et al. (2021) who found an increase in ascorbic acid and polyphenols of carrot microgreens and an opposite trend in dill. In general, both biostimulants act via different ways, increasing the plant metabolic efficiency and promoting the production of secondary metabolites which protect the plant from oxidative stress. Specifically, amino acids and peptides comprised in PH can stimulate the synthesis of antioxidant enzymes, such as ascorbate peroxidase, which increases the production of ascorbic acid (Pasković et al., 2024). It was reported that the PH application increases plant defense via the enhancement of secondary metabolism activity to produce antioxidant compounds, like phenols and flavonoids (Zhang et al., 2023). SWE can promote the biosynthesis of ascorbic acid, phenols and flavonoids via different mechanisms: i) SWE application enhances nutrient uptake, which in turn boosts the production of ascorbic acid (Abbas et al., 2020) and ii) SWE comprises several phyto-compounds that boost the activity of enzymes, like flavonoid synthase and Phenylalanine Ammonium-Liasis (PAL), linked to the phenols and flavonoids biosynthesis (Zewail, 2014).

Total sugars are an important parameters since they contribute to nutritional and sensory characteristics of microgreens. In our research, pea total sugars were affected by biostimulants application. The recorded data on pea might suggested that the efficacy of biostimulants on carbohydrate metabolism primary depends on species physiological traits. These data are consistent with those of Schiavon et al. (2008) and of Shehata et al. (2011). Protein hydrolysates contain peptides and hormones capable of stimulating photosynthesis activity and, accordingly, the production of organic molecules like sugars thanks to carbon fixation (Sun et al., 2024). Furthermore, there is evidence that PH applications may boost the activity of enzymes, like amylase, invertase, and sucrose synthase, directly involved in sugars production (Pasković et al., 2024). Similar mechanisms can be ascribed to SWE since it contains plant hormones (auxin, cytokinin and gibberellin) that upsurge the photosynthesis rate in treated plants (Ali et al., 2021). Cytokinin can promote the accumulation of sugars via the modulation of carbohydrate flow in plants (Roitsch and Ehneß, 2000). Additionally, both biostimulants can interfere with sugars transport, accumulation and distribution in plants (Kumar et al., 2020; Malécange et al., 2023). Our study also pointed out that biostimulants effect on total sugars was only significant in pea microgreens and only when PH and SWE were supplied via root application, underlining that the response can be influenced by the species and by the type of application. This suggests that the application via priming is not enough for eliciting plant responses to biostimulants application in the total sugar’s modulation and confirm our hypothesis regarding the importance of continuous application of the biostimulant in order to achieve certain positive effects on the plant traits.

Biostimulants significantly improved total chlorophyll and carotenoids concentration in both species. These findings are in accordance with those reported by Asadi et al. (2022) who found an increase in chlorophyll and carotenoids when SWE was administered on lettuce plants. Furthermore, our data concur with those of El-Nakhel et al. (2021) who reported an increase in chlorophyll and carotenoids of carrot and dill microgreens treated with PH. At this regard, it was reported that amino acids, such as glycine and glutamic acid, comprised in PH can stimulate the biosynthesis of photosynthetic pigments, such as chlorophyll and carotenoids (Noroozlo et al., 2019). Furthermore, nutrients and hormones enclosed in SWE can stimulate the biosynthesis of pigments of treated plants via the elicitation of metabolism (Sunarpi et al., 2011). Our research also showed that priming significantly boosted chlorophyll and carotenoids concentration of pea and arugula microgreens. These data concur with those of Ciriello et al. (2023) on mibuna and komatsuna, while are in contradiction with those of Ciriello et al. (2024). Since priming helps plants to cope with environmental stress (UV radiation, non-optimal temperature, drought, etc.) (Jisha et al., 2013), we can speculate that the production of carotenoids is one of the mechanisms by which priming ameliorated plant stress tolerance. As reported by Zhu et al. (2021), priming can also alter the biosynthesis of plant hormones, like auxin and cytokinin, involved in the production of photosynthetic pigments. Priming treatment can also activate genetic and epigenetic mechanisms, influencing chlorophyll and carotenoids concentration in plant (Turgut-Kara et al., 2020). Additionally, the higher growth of primed plants favors a more active photosynthetic system, with positive outcomes on carotenoids production (Anwar et al., 2020).

Root application treatments and biostimulant application enhanced total chlorophyll/carotenoids ratio in *E. sativa*. These outcomes are in line with those of Toscano et al. (2023) who revealed a reduction in the chlorophyll/carotenoids ratio when PH was supplied on turnip, whereas no significant effect on radish was recorded. These contrasting outcomes underlined the different responses of species in the modulation of chlorophyll/carotenoids ratio. The chlorophyll/carotenoids ratio is an important plant health indicator since it explains the relationship between the two principal photosynthetic pigments (Shah et al., 2017). If the ratio is high, as we observed in response to the root application of PH/SWE treatments (**Table 2**), it indicated that plants have a good photosynthetic activity, which in turn suggests its efficacy in light capture. Since carotenoids protect cells from oxidative stress, a lower ratio suggests that plants react to stress or, as we may assume in this study, can cope with environmental stress due to priming or biostimulants application. To further confirm these data, the values of DPPH scavenging increased in biostimulated plants. Even though the type of application had no significant effect on DPPH, a decreasing trend in primed plants was observed.

The economic assessment showed that the added net return gained using biostimulants highly depends on the microgreen’s species and biostimulant employed. Particularly, for pea, the added net return was always positive, indicating an economic convenience for biostimulants application, independently of the type of application, with peaks in hydropriming and PH priming treatments. Inversely, for arugula, the treatments PH priming and SWE root application gave negative added net return. Generally, the added variable cost for both species were very low due to the similar harvest and application cost and to the low quantity of biostimulant used. The recorded differences might be attributed to the different growth rate of the two species, which in turn affected the plant response to biostimulant application. However, for pea microgreens performance, a predominant effect of the priming was recorded. These results pointed out that the application of biostimulants is not always economically beneficial, but it cost-effectiveness highly depends on the combination of species, biostimulants and its type of application.

## 5 Conclusion

In our study, we investigated the effect of two biostimulants, a plant protein hydrolysate and a seaweed extract, administered via priming or root application on pea and arugula microgreens. Biostimulants enhanced the concentration of P, K, ascorbic acid, total phenols, flavonoids, total chlorophyll and DPPH in both species compared to the control. Priming increased pea yield, however had no significant effect on arugula yield. Priming elicited the production of flavonoids and carotenoids. Nitrate concentrations were enhanced by SWE and reduced by PH in arugula microgreens. Our research suggested that the application of biostimulants via priming potentially enhanced ascorbic acid, total phenols, flavonoids and carotenoids, whereas root application supply meaningfully boosted mineral concentrations, total sugars, total chlorophyll and total chlorophyll/carotenoids ratio. This study also showed that nitrate concentration was enhanced by SWE and reduced by PH in arugula microgreens. Furthermore, the economic estimation proposed that the use of both biostimulants, independently of the type of application, is economically convenient for pea, but not suitable for arugula when PH via priming or SWE via root application were employed (negative returns).

Although the study demonstrated the potential of biostimulants for improving the performance of arugula and pea microgreens, it could be useful to extend the treatments to other vegetables. However, this study lays the foundations for further investigations, including omics studies, useful to explore the mechanisms involved.

## Data Availability

The raw data supporting the conclusions of this article will be made available by the authors, without undue reservation.
