# Supplementary material for: Combining non-microbial biostimulants and different application methods affects yield, antioxidant activity, nutritional and functional value of pea and arugula microgreens
Source: Front Plant Sci. 2026 Aug 31;17:1913861. doi: 10.3389/fpls.2026.1913861 (PMC13572118; doi:10.3389/fpls.2026.1913861)
Supplement: Supplementary file 1 [file Table1.docx]

**Table S1.** Aminogram of Tyson® protein hydrolysate.

| **Amino acids** | **% W/W** |
| --- | --- |
| Alanine | 0.83 |
| Arginine | 0.11 |
| Aspartic acid | 0.72 |
| Glutamic acid | 7 |
| Glycine | 0.05 |
| Lysine | 0.05 |
| Methionine | 0.05 |
| Phenylalanine | 0.05 |
| Proline | 3.21 |
| Tryptophan | 1.25 |
| Valine | 0.05 |

**Table S2.** Shapiro-Wilk and Levene tests for pea data.

| **Parameters** | **Shapiro-Wilk** | | | **Levene** | | | |
| --- | --- | --- | --- | --- | --- | --- | --- |
|  | **W** | **df** | **Sig.** | **F** | **df1** | **df2** | **Sig.** |
| Yield | 0.971 | 18 | 0.812 | 0.769 | 5 | 12 | 0.590 |
| Dry matter | 0.932 | 18 | 0.210 | 1.803 | 5 | 12 | 0.187 |
| Nitrate | 0.968 | 18 | 0.768 | 2.578 | 5 | 12 | 0.083 |
| P | 0.969 | 18 | 0.773 | 1.308 | 5 | 12 | 0.324 |
| K | 0.957 | 18 | 0.537 | 1.019 | 5 | 12 | 0.449 |
| Ca | 0.946 | 18 | 0.364 | 0.844 | 5 | 12 | 0.544 |
| Mg | 0.971 | 18 | 0.823 | 0.826 | 5 | 12 | 0.555 |
| Ascorbic acid | 0.952 | 18 | 0.464 | 0.892 | 5 | 12 | 0.516 |
| Total phenols | 0.930 | 18 | 0.197 | 2.025 | 5 | 12 | 0.147 |
| Flavonoids | 0.927 | 18 | 0.174 | 0.935 | 5 | 12 | 0.493 |
| Total sugars | 0.968 | 18 | 0.754 | 1.330 | 5 | 12 | 0.316 |
| Total chlorophyll | 0.983 | 18 | 0.977 | 1.331 | 5 | 12 | 0.316 |
| Carotenoids | 0.896 | 18 | 0.048 | 2.259 | 5 | 12 | 0.115 |
| Total chlorophyll/carotenoids ratio | 0.928 | 18 | 0.180 | 2.440 | 5 | 12 | 0.095 |
| DPPH | 0.963 | 18 | 0.667 | 0.886 | 5 | 12 | 0.519 |

**Table S3.** Shapiro-wilk and Levene tests for arugula.

| **Parameters** | **Shapiro-Wilk** | | | **Levene** | | | |
| --- | --- | --- | --- | --- | --- | --- | --- |
|  | **W** | **df** | **Sig.** | **F** | **df1** | **df2** | **Sig.** |
| Yield | 0.972 | 18 | 0.826 | 2.250 | 5 | 12 | 0.116 |
| Dry matter | 0.942 | 18 | 0.313 | 2.795 | 5 | 12 | 0.067 |
| Nitrate | 0.981 | 18 | 0.963 | 1.145 | 5 | 12 | 0.389 |
| P | 0.979 | 18 | 0.935 | 0.938 | 5 | 12 | 0.491 |
| K | 0.947 | 18 | 0.382 | 0.904 | 5 | 12 | 0.510 |
| Ca | 0.981 | 18 | 0.956 | 1.633 | 5 | 12 | 0.225 |
| Mg | 0.967 | 18 | 0.741 | 2.388 | 5 | 12 | 0.101 |
| Ascorbic acid | 0.968 | 18 | 0.755 | 1.507 | 5 | 12 | 0.259 |
| Total phenols | 0.935 | 18 | 0.240 | 2.603 | 5 | 12 | 0.081 |
| Flavonoids | 0.966 | 18 | 0.725 | 1.838 | 5 | 12 | 0.180 |
| Total sugars | 0.922 | 18 | 0.138 | 1.112 | 5 | 12 | 0.404 |
| Total chlorophyll | 0.940 | 18 | 0.286 | 0.703 | 5 | 12 | 0.632 |
| Carotenoids | 0.948 | 18 | 0.400 | 2.368 | 5 | 12 | 0.103 |
| Total chlorophyll/carotenoids ratio | 0.886 | 18 | 0.033 | 0.686 | 5 | 12 | 0.643 |
| DPPH | 0.941 | 18 | 0.300 | 0.684 | 5 | 12 | 0.645 |
